# ROCK Inhibition Facilitates *In Vitro* Expansion of Glioblastoma Stem-Like Cells

**DOI:** 10.1371/journal.pone.0132823

**Published:** 2015-07-13

**Authors:** Samantha G. Tilson, Elizabeth M. Haley, Ursula L. Triantafillu, David A. Dozier, Catherine P. Langford, G. Yancey Gillespie, Yonghyun Kim

**Affiliations:** 1 Department of Chemical and Biological Engineering, The University of Alabama, Tuscaloosa, Alabama, United States of America; 2 Department of Neurosurgery, The University of Alabama at Birmingham, Birmingham, Alabama, United States of America; University of Alabama at Birmingham, UNITED STATES

## Abstract

Due to their stem-like characteristics and their resistance to existing chemo- and radiation therapies, there is a growing appreciation that cancer stem cells (CSCs) are the root cause behind cancer metastasis and recurrence. However, these cells represent a small subpopulation of cancer cells and are difficult to propagate *in vitro*. Glioblastoma is an extremely deadly form of brain cancer that is hypothesized to have a subpopulation of CSCs called glioblastoma stem cells (GSCs; also called brain tumor initiating cells, BTICs). We propose the use of selective Rho-kinase (ROCK) inhibitors, Y-27632 and fasudil, to promote GSC/BTIC-like cell survival and propagation *in vitro*. ROCK inhibitors have been implicated in suppressing apoptosis, and it was hypothesized that they would increase the number of GSC/BTIC-like cells grown *in vitro* and improve cloning efficiencies. Indeed, our data demonstrate that transient and continuous supplementation of non-toxic concentrations of Y-27632 and fasudil inhibited apoptosis, enhanced the cells’ ability to form spheres, and increased stem cell marker expressing GSC/BTIC-like cell subpopulation. Our data indicated that pharmacological and genetic (siRNA) inhibitions of the ROCK pathway facilitates *in vitro* expansion of GSC/BTIC-like cells. Thus, ROCK pathway inhibition shows promise for future optimization of CSC culture media.

## Introduction

Glioblastoma (GBM) is the deadliest form of brain cancer. Patients diagnosed with glioblastoma have poor prognosis, and their median survival rate is approximately one year [1]. Recent research shows evidence for a specialized subpopulation of glioblastoma cells called glioblastoma stem cells (GSCs; also called brain tumor-initiating cells, BTICs) [2, 3]. GSCs/BTICs have the ability to self-renew and differentiate into the heterogeneous tumor cells that constitute the entire tumor. It is hypothesized that a single GSC has the ability to form an entire bulk tumor, so these cells are implicated in metastasis and tumor regeneration after treatment [4]. GSCs are also thought to be resistant to current treatment techniques such as chemotherapy and radiation [3, 5–9]. For these reasons, the study of GSCs is an area of interest in current glioblastoma research.

It would be ideal to develop *in vitro* GSC/BTIC-like models as animal models are costly, time-, and labor-consuming. However, GSCs are notoriously difficult to culture in conventional *in vitro* conditions, and many have often questioned whether the cells grown *in vitro* are a true representation of the cancer found *in vivo*. Cells grown *in vitro* experience mechanical stresses that they would not experience natively *in vivo*, such as shear stress from trituration during passaging. Trituration is a mechanical method for dissociating cell aggregates by applying shear stress to the cells with a pipette tip. This shear stress can be very harmful and even fatal to the cells, which makes retaining high cell yields during passaging very difficult. After several passages *in vitro*, cells may also undergo genetic mutations that alter their characteristics and behavior to help them survive the stresses of the new *in vitro* environment [10]. This is one of many reasons that the results of *in vitro* studies often do not translate well to those found in pre-clinical and clinical studies. We and others are developing technologies to better mimic *in vivo* conditions to decrease the population of mutated cells and to improve the validity and success of studies performed *in vitro*. When glioblastoma cells are grown in serum-free media, they form tumorspheres which were previously shown to better imitate the tumor niche *in vivo* and are enriched for GSC/BTIC-like cells [11]. However, these tumorspheres must be dissociated frequently for further propagation *in vitro*. We hypothesized that treatment with ROCK inhibitors would enhance the cells’ ability to survive the shear stress of trituration while maintaining characteristics of the original tumor.

Y-27632 and fasudil are known inhibitors to the ROCK pathway [12]. The ROCK pathway is active in several cellular events including apoptosis and actin stress fiber formation [13]. They have previously been shown to inhibit dissociation-induced apoptosis in human embryonic stem cells and other stem cells that are susceptible to anoikis [14–20]. We hypothesized that the addition of either ROCK inhibitor to the culture media would help GSC-like cells survive the stresses of *in vitro* dissociation. In addition, it was also hypothesized that the inhibition of apoptosis via ROCK inhibitors would increase the total number of cells as well as the total number of GSC-like cells.

Here, we study the effects of Y-27632 and fasudil on the *in vitro* expansion of GSC/BTIC-like cells. We demonstrate that these inhibitors are nontoxic and in some cases, improve cells’ metabolic activity and viability. Our data suggest that Y-27632 and fasudil inhibit apoptosis in cultures and increase the total number of cells. Using flow cytometry and limiting dilution assays, we show that the ROCK inhibitors increase the concentration of GSC-like cells in culture. ROCK inhibitors thus promise to be a valuable addition to the culture media that protects the GSC-like cells from apoptosis from dissociation- and passaging-induced shear stress in *in vitro* culture.

## Materials and Methods

### Cell Culture

Three glioblastoma cell lines were used: U87-MG (ATCC, Manassas, VA), primary glioblastoma cell line SMC448 (kindly provided by Dr. Do-Hyun Nam, Samsung Medical Center, Seoul, South Korea), and JX12. JX12 is a classical subtype patient-derived GBM xenograft cell line (xenoline) that was established as previously described [21] in immunocompromised athymic nude mice from surgical resection waste specimens obtained from consented patient undergoing surgical therapy for primary GBM at the University of Alabama at Birmingham Comprehensive Cancer Center Brain Tumor Tissue Core Facility under the approval of annually renewed IRB (approval no. X050415007). The cells were grown in three-dimensional tumorsphere culture in Neurobasal media supplemented with 1 mM glutamine (Life Technologies, Carlsbad, CA), 8 μg/mL heparin (JT Baker, Phillipsburg, NJ), 0.5X N2 (Gibco, Grand Island, NY), 0.5X B27 (Gibco), 1% Penicillin/Streptomycin (Corning, Manassas, VA), 20 ng/mL EGF (Shenandoah Inc., Warwick, PA), and 10 ng/mL FGF (Shenandoah Inc) (NBE media). For the Y-27632 and fasudil experimental groups, NBE was supplemented with either 45 μM Y-27632 (Thermo Fisher Scientific, Pittsburg, PA) or 10 μM fasudil hydrochloride (Biotang Inc., Lexington, MA), respectively.

### Toxicity Assay

The relative toxicity of five log concentrations of Y-27632 and fasudil (0.1 μM, 1 μM, 10 μM, 100 μM, and 1000 μM) were tested. U87-MG, JX12, and SMC448 cells were seeded in 96 well plates at a seeding density of 1x10^4^ cells/well (*n* = 10 for each ROCK inhibitor). The resulting cell viability was measured at 450 nm absorbance using a water-soluble tetrazolium salt-based proliferation assay according to manufacturer’s protocol (Cell Counting Kit-8, Enzo Life Sciences, Farmingdale, NY). The data were normalized to those of the control (group not treated with either inhibitor) to measure relative cell viability.

### Sphere Analysis

Cells were cultured for six days in control media and in media supplemented with either 45 μM Y-27632 or10 μM fasudil. Micrographs were taken (*n* = 20) of each experimental group at 4X magnification throughout the culture period. The number of spheres was counted in each micrograph. The diameter of each tumorsphere was measured using ImageJ (National Institutes of Health, Bethesda, MD). Statistical analyses were performed in Minitab v16 (Minitab Inc., State College, PA).

### Flow Cytometry

To measure apoptosis using flow cytometry, U87-MG, JX12, and SMC448 cultured in NBE with 45 μM Y-27632, with 10 μM fasudil, or without either inhibitor (control) were stained with Annexin V (Enzo Life Sciences) and propidium iodide (EMD Millipore, Billerica, MA). To measure the GSC population, cells were permeabilized with Permeablization Kit (R&D Systems, Minneapolis, MN), stained with mouse IgG anti-human SOX2 primary antibody (Abgent, San Diego, CA), and anti-mouse IgG secondary antibody conjugated with fluorescein isothiocynate (R&D Systems). All flow cytometry was performed using BD Accuri C6 Flow Cytometer (BD Biosciences, San Jose, CA).

### Limiting Dilution Assay

Limiting dilution assay (LDA) was performed as previously described [22–24]. For all experimental groups (control, 45 μM Y-27632, 10 μM fasudil), cells were seeded in 96 well plates with varying cell densities of 1–20 cells/well (*n* = 20 wells for each seeding density). Cells were cultured for two weeks with supplemental feeding with appropriate media every three days. On Day 14, wells with tumorspheres were counted. Spheres were defined as cell aggregates of at least 30 μm in diameter. IBM SPSS v22 was used for statistical analyses (Armonk, NY).

### RNA Interference

For small interfering RNA (siRNA)-mediated knockdown of *ROCK2* (NM_004850), cells were transfected with 100 nM of either the targeting or control siRNA (Sigma-Aldrich, St. Louis, MO) using Lipofectamine RNAiMAX (Life Technologies) for 72 hours according to manufacturer’s protocol. Three independent *ROCK2*-targeting siRNAs were used (Sigma-Aldrich MISSION siRNA; labeled as siRNA-1, siRNA-2, siRNA-3).

### qRT-PCR

Primers were designed by retrieving nucleotide sequences from NCBI gene database for *ROCK2* (NM_004850), *CASP3* (NM_004346), and *CASP7* (NM_001227). Primers used include *ROCK2* forward: TTT CGT ACA GGC AAT GAA AGC C; *ROCK2* reverse: GGA GAA TTG TTA TCT TTA GCC TCA C; *CASP3* forward: GCG AGC ACT CAC GAA ACT CT; *CASP3* reverse: TAT CCC GGG TTG ACA ATG TGG; *CASP7* forward: AGG TTT GCA CAG GTT CTT GC; and *CASP7* reverse: AGA CTC CCA GTG GTT GCT TT. *GAPDH* (NM_001256799) was used as a housekeeping gene, with the primer sequence for *GAPDH* forward: AGA GCA CAA GAG GAA GAG AGA GAC and *GAPDH* reverse: AGC ACA GGG TAC TTT ATT GAT GGT. Primers were synthesized by Eurofins Genomics (Huntsville, AL).

RNA isolation was performed using GeneJet RNA Purification Kit (Thermo Fisher Scientific) according to manufacturer’s protocol for mammalian cultured cells. RNA quantification was performed using Qubit RNA HS Assay Kit and Qubit 2.0 Fluorometer (Life Technologies). Complementary DNA (cDNA) was synthesized using qScript cDNA SuperMix (Quanta Biosciences, Gaithersburg, MD) and Mastercycler nexus gradient (Eppendorf, Hauppauge, NY) according to manufacturers’ protocols. Quantitative real-time PCR was performed using PerfeCTa SYBR Green FastMix (Quanta Biosciences) according to manufacturer’s protocol. Illumina Eco was used as the qRT-PCR instrument along with EcoStudy software for the data analysis.

### Statistical Analyses

All statistical analyses were performed on Minitab or SPSS. For comparison of sphere diameters and sphere numbers, Student’s *t*-test or ANOVA with Tukey’s HSD post hoc analysis was performed on Minitab. For limiting dilution assays, general linear model was used to generate the linear regressions and to compare the resulting slopes via SPSS. With all these analyses, significance was set at *p* < 0.05.

## Results

### ROCK inhibitors are not toxic to GBM tumorspheres at low concentrations

**Fig 1:**
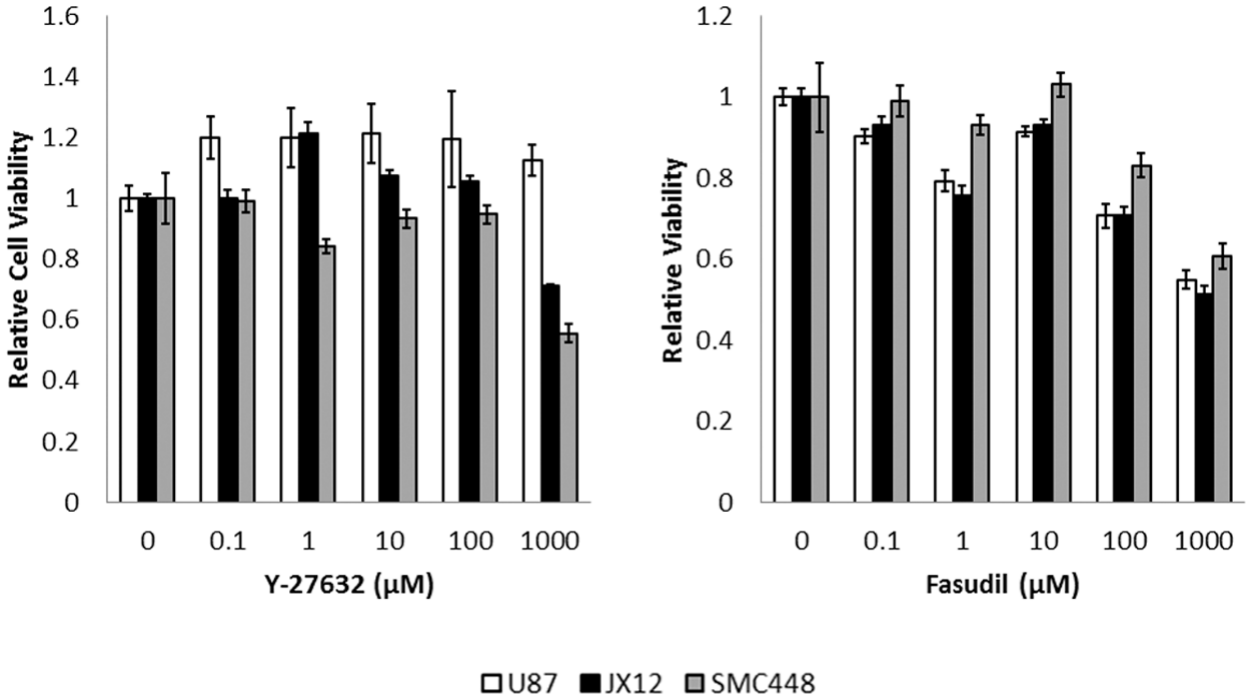
ROCK inhibitors are not toxic to GBM tumorspheres at low concentrations. The toxicities of Y-27632 and fasudil were measured using a water-soluble tetrazolium assay (WST-8 Cell Counting Kit 8). U87-MG, JX12, and SMC448 cells were exposed to varying concentrations of Y-27632 or fasudil for 48 hours. Cell viability was measured relative to 0 μM control (*n* = 10).

Cell viability was measured using a water-soluble tetrazolium assay to test whether the Rho kinase inhibitors Y-27632 and fasudil had any toxic effects on established and primary GBM cell lines grown as tumorspheres (Fig 1). Forty-eight hour exposure to the inhibitor did not display toxic effects on any of the cell lines in the tested five log concentration range. Y-27632 had a generally positive effect on cell viability in JX12 and U87-MG compared to the control (0 μM). The cells displayed maximum metabolic activity at Y-27632 concentrations between 1 and 100 μM. Y-27632 did not become inhibitory to the glioblastoma tumorspheres until extremely high concentrations (>1000 μM). For this reason, we chose to supplement with 45 μM of Y-27632 for our subsequent experiments, which is also comparable to previously published reports [25]. The cells were slightly more sensitive to the addition of fasudil. The cells reached maximum metabolic activity at a concentration of 10 μM fasudil and the inhibitor began to hinder growth at a concentration of 100 μM fasudil. Therefore, 10 μM fasudil was chosen as the concentration for subsequent tests.

### ROCK inhibitors protect GBM tumorspheres from apoptosis

**Fig 2:**
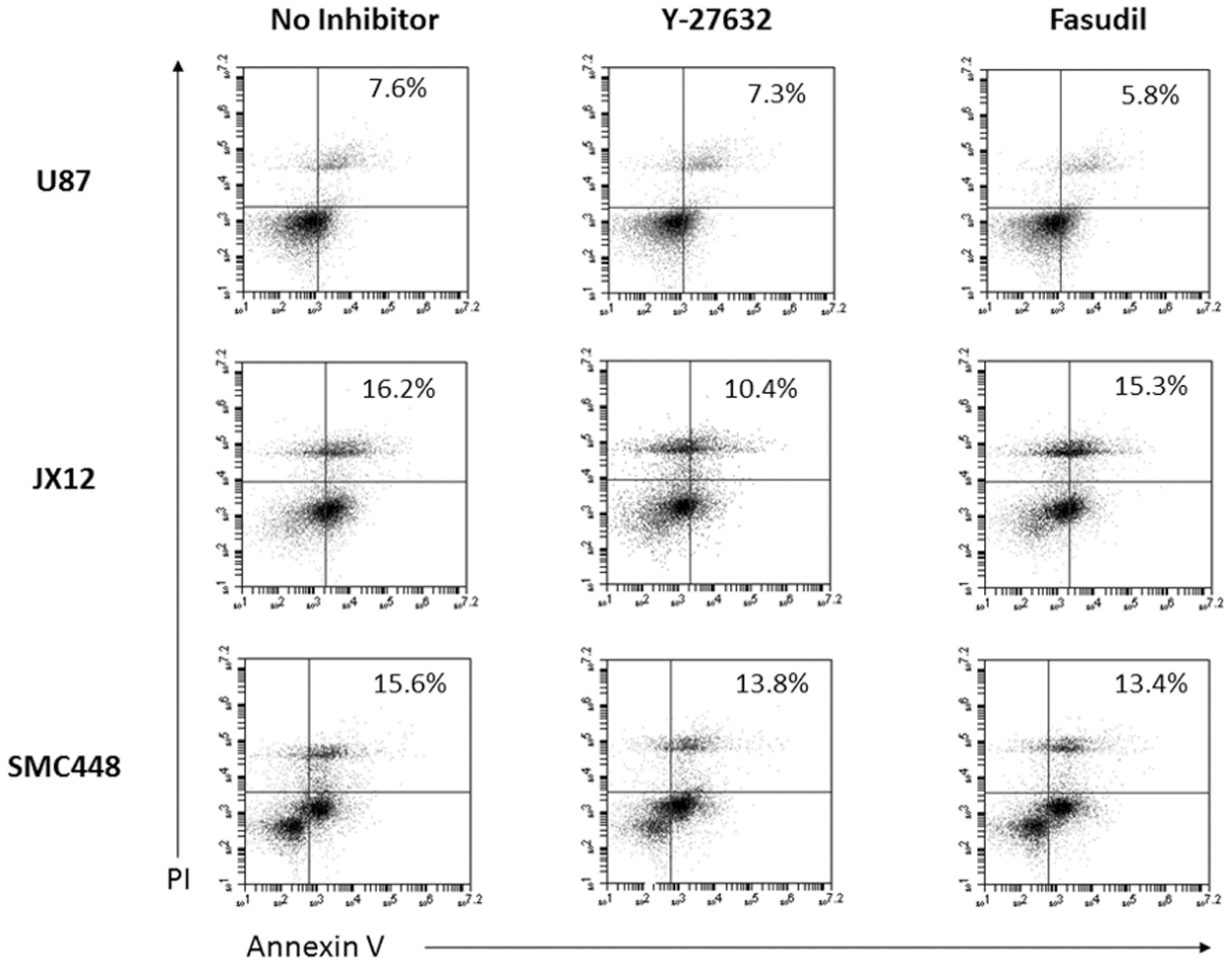
ROCK inhibitors protect GBM tumorspheres from apoptosis. Flow cytometry was used to quantify the late-stage apoptotic cells (Annexin V^+^/PI^+^) immediately after trituration. The cells that were treated with 45μM Y-27632 or 10 μM fasudil had decreased number of late-stage apoptotic cells in U87-MG, JX12, and SMC448 cell lines, indicating that the ROCK inhibitors Y-27632 and fasudil inhibited apoptosis in glioblastoma cells.

*In vitro* culture processing can be inherently stressful for tumorspheres, especially when exposed to the shear stress of trituration necessary for single cell dissociation. Our data indicated that treatment with Y-27632 and fasudil helped more cells survive the stresses of cell culture (Fig 2). Using flow cytometry, treatment with Y-27632 or fasudil was found to decrease the Annexin V/propidium iodide (PI) double-positive population for all three cell lines, signifying the decreased number of cells in late-stage apoptosis. These results are consistent with prior reports that showed that the ROCK pathway is active in membrane blebbing, an early stage of apoptosis [13]. Our data therefore imply that ROCK pathway inhibition by Y-27632 and fasudil protected the GBM tumorspheres from pre-mature apoptosis.

### ROCK inhibitors enhance GBM tumorsphere formation

**Fig 3:**
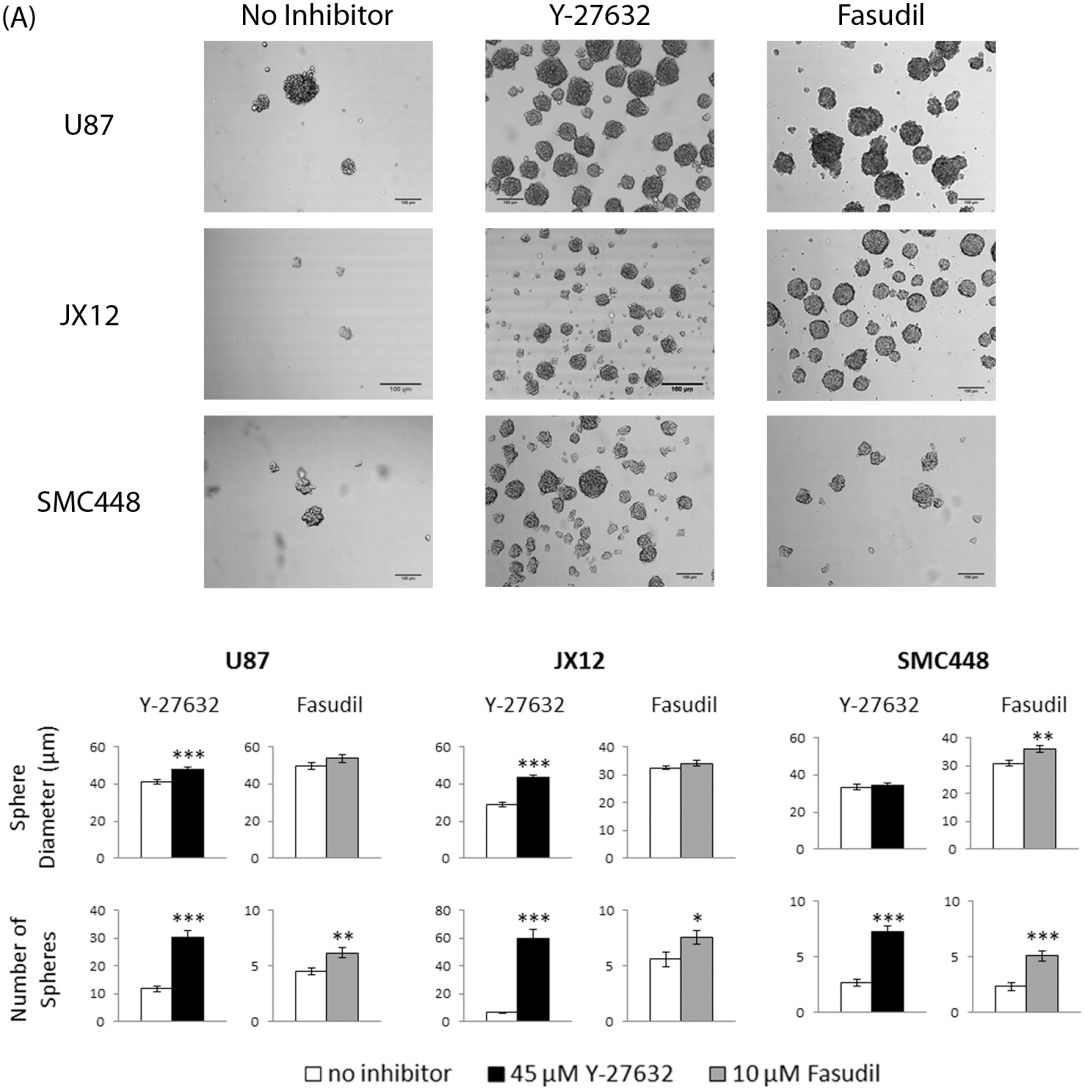
ROCK inhibitors enhance GBM tumorsphere formation. The glioblastoma cells were grown as tumorspheres in two concentrations of Y-27632 (0 and 45 μM) or two concentrations of fasudil (0 and 10μM) for 6 days. (A) Sample micrographs of each experimental group on Day 6 (Scale bars = 100 μm). (B) The sphere diameter and number of spheres were analyzed for all experimental groups on Day 3 (mean ± SE; *n* = 100). It was found that the sphere diameter stayed relatively consistent between the experimental groups. The number of spheres per field of view in each experimental group were also quantified from the micrographs (mean ± SE; *n* = 20; * *p* < 0.05, ** *p* < 0.01, and *** *p* < 0.001).

The ROCK pathway is also involved in the formation of actin stress fibers that participate in cell-to-cell adhesion [12, 13, 26]. Previous reports demonstrated that tumorsphere formation is an essential hallmark of enriching GSCs *in vitro* [11, 27]. Therefore, we hypothesized that inhibition of the ROCK pathway via Y-27632 or fasudil may disrupt the cell-to-cell adhesion necessary for tumorsphere formation. Quantitative analysis of tumorsphere formation and their diameter was performed on U87-MG, JX12, and SMC448 cells cultured in the presence and absence of Y-27632 and fasudil (0 μM and 45 μM; 0 μM and 10μM; *n* = 100 spheres per group; Fig 3). In contrast to our hypothesis, the tested concentrations of Y-27632 and fasudil did not hinder tumorsphere formation but rather enhanced it. Furthermore, the sphere diameter in each experimental group was similar to or larger than the control in all three cell lines, indicating that the inhibition of cell-to-cell adhesion by the ROCK inhibitors was minimal at the tested concentrations with GBM tumorspheres. In addition, the number of spheres in each experimental group significantly increased (*p* < 0.05) with the addition of Y-27632 and fasudil. Taken together with previous analysis of apoptosis inhibition, the increase in the number of GBM tumorspheres suggested that Y-27632 and fasudil functionally provide pro-survival and pro-tumorsphere formation mechanisms.

### ROCK inhibitors enhance stem-like phenotype

**Fig 4:**
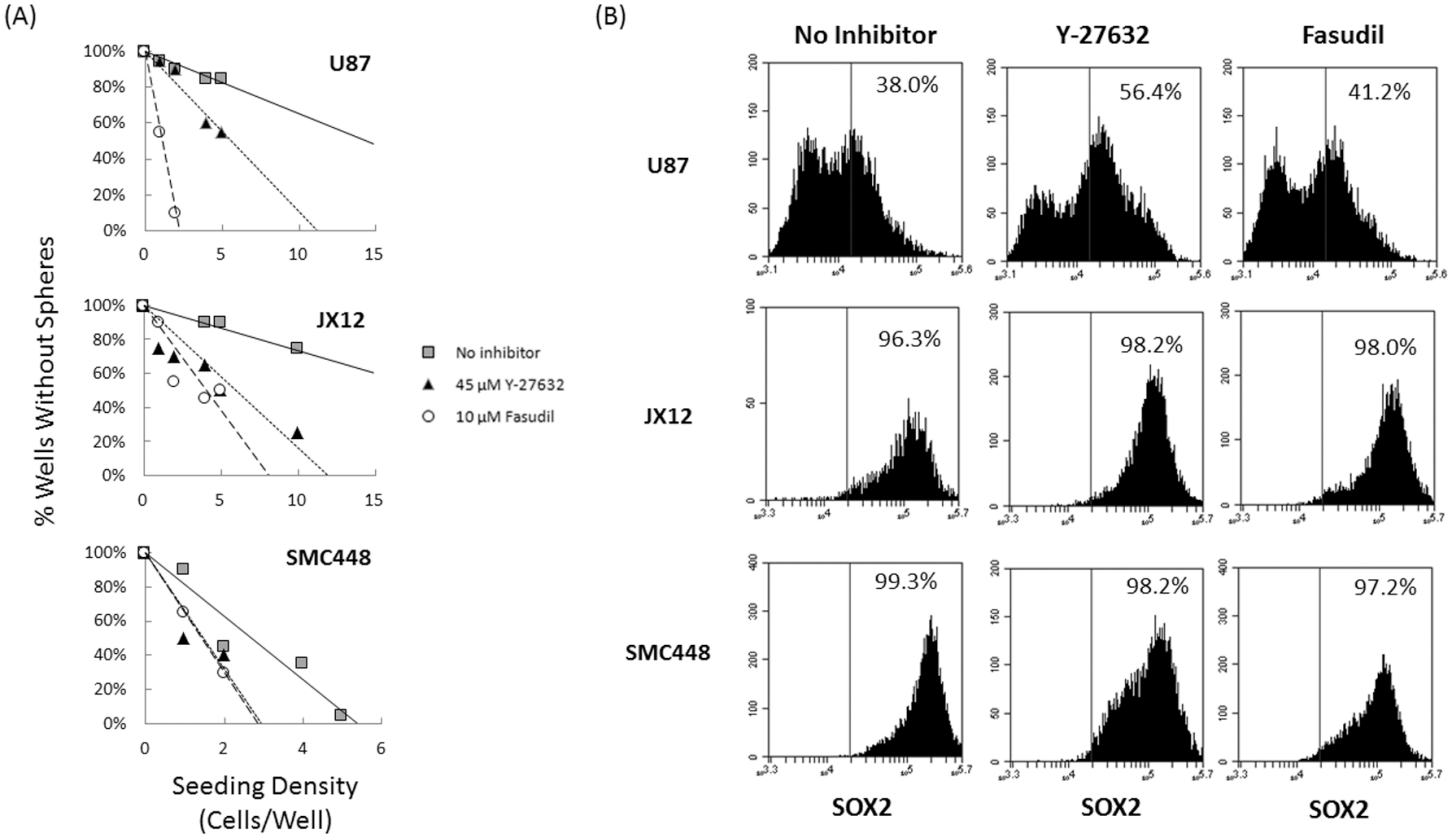
ROCK inhibitors enhance GSC-like stemness. (A) The clonogenicity of the cells was quantified using limiting dilution assay (steeper slope and lower value in x-intercept indicates increased clonogenic potential and stemness). The cells treated with 45 μM Y-27632 or 10 μM fasudil required fewer cells to form spheres indicating increased number of GSC-like cell than control. (B) The glioblastoma cells were grown as tumorspheres in two concentrations of Y-27632 (0 and 45μM) or two concentrations of fasudil (0 and 10 μM) for three days. Using flow cytometry, the percentage of the total population expressing the GSC marker SOX2 was quantified.

Since increased tumorsphere formation is linked with GBM stemness, the role of Y-27632 and fasudil in stem cell marker expression and self-renewal was investigated. Expression levels of the previously reported GSC marker SOX2 were tested. Our data indicated that the presence of Y-27632 and fasudil increased the subpopulation that possessed the SOX2 GSC marker in U87-MG and JX12; however, there was a slight decrease in SOX2 expression in SMC448, albeit it was already highly expressing (Fig 4). In addition, the limiting dilution assay (LDA), an *in vitro* assay that measures clonogenicity, revealed that cells grown with Y-27632 or fasudil had increased self-renewal potential and more readily formed tumorspheres, indicating an increased GSC/BTIC-like subpopulation.

### Transient treatment with ROCK inhibitors is sufficient to promote GSC/BTIC-like cell expansion

**Fig 5:**
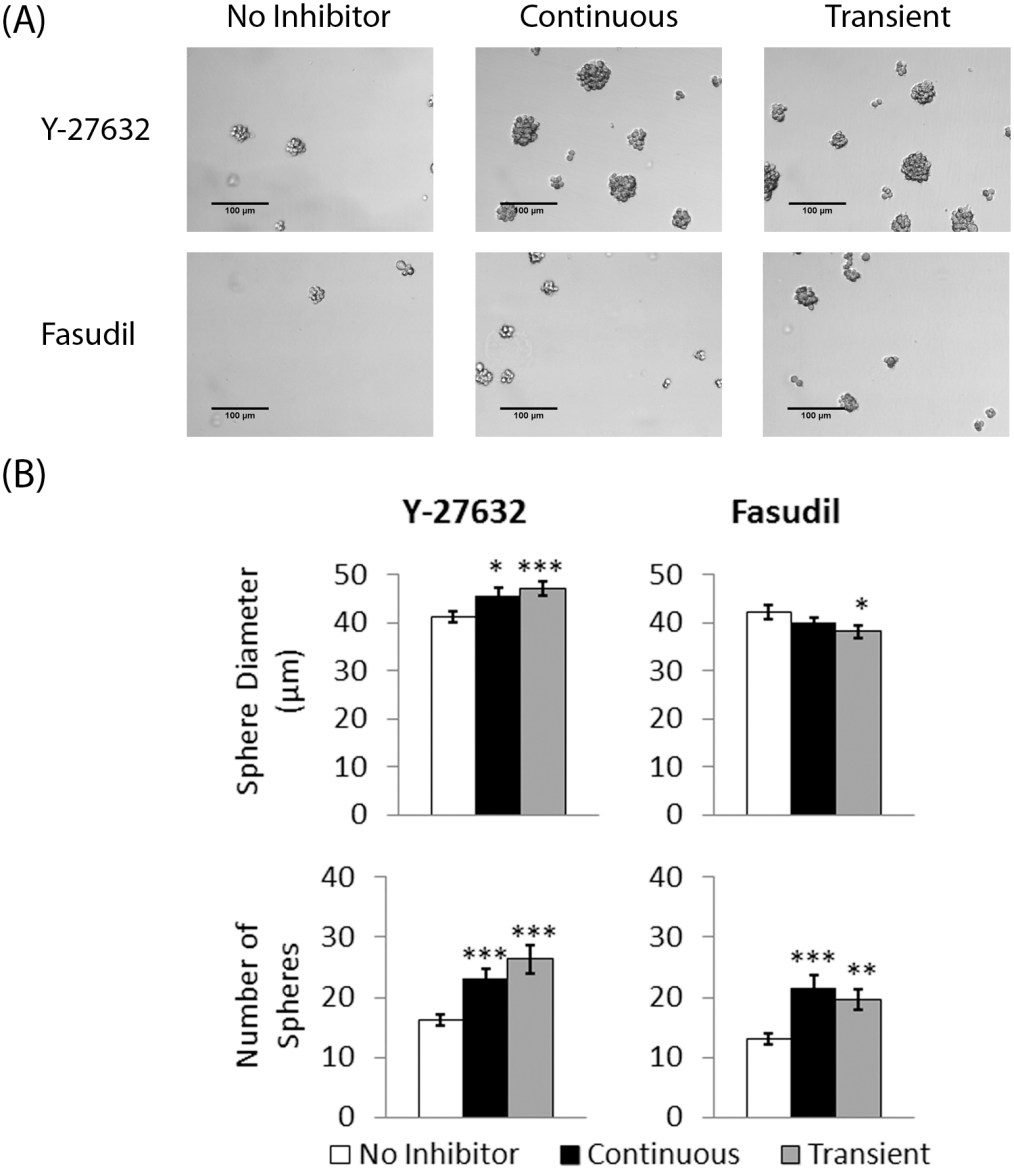
Transient treatment with ROCK inhibitors is sufficient to promote GSC-like cell expansion. The cells were treated with no inhibitor, with continuous exposure to the ROCK inhibitor (45 μM Y-27632 or 10 μM fasudil), or with transient exposure to the ROCK inhibitor (45 μM Y-27632 or 10 μM fasudil). (A) Representative micrographs of each experimental group on Day 3 (Scale bars = 100 μm). (B) The sphere diameter and number of spheres were analyzed for all experimental groups on Day 3 (mean ± SE; *n* = 100; * *p* < 0.05, ** *p* < 0.01, and *** *p* < 0.001). The number of spheres per field of view in each experimental group were also quantified from the micrographs (mean ± SE; *n* = 20; * *p* < 0.05, ** *p* < 0.01, and *** *p* < 0.001).

The effects of transient treatment of ROCK inhibitors on GBM cells were investigated to compare our findings with previous reports that successfully inhibited apoptosis in embryonic stem cells treated with ROCK inhibitors for only two to six hours [14, 15, 24]. U87-MG cells were treated with 45 μM Y-27632 or 10 μM fasudil for four hours during passaging into fresh NBE media. Sphere formation analysis was performed on these treated cells and compared to those continually exposed to the inhibitors and to those grown in control media (NBE media with no ROCK inhibitors). After three days, both the cells that were continually treated with the ROCK inhibitors and the cells that were transiently treated with the ROCK inhibitors showed enhanced sphere formation ability (Fig 5). The cells exposed to Y-27632, both continually and transiently, showed a significantly higher sphere diameter (*p <* 0.05 and *p* < 0.001, respectively) and number of spheres compared to the control (*p* < 0.001 for both). For the cells exposed to fasudil, there was no significant difference in sphere diameter but there were significantly more spheres compared to the control (*p <* 0.001 and *p <* 0.05, respectively). These data indicate that transient treatment with ROCK inhibitors during passaging is sufficient to promote enhanced expansion of GSC-like cells.

### Knockdown of ROCK2 shows similar behavior to Y-27632 and Fasudil

**Fig 6:**
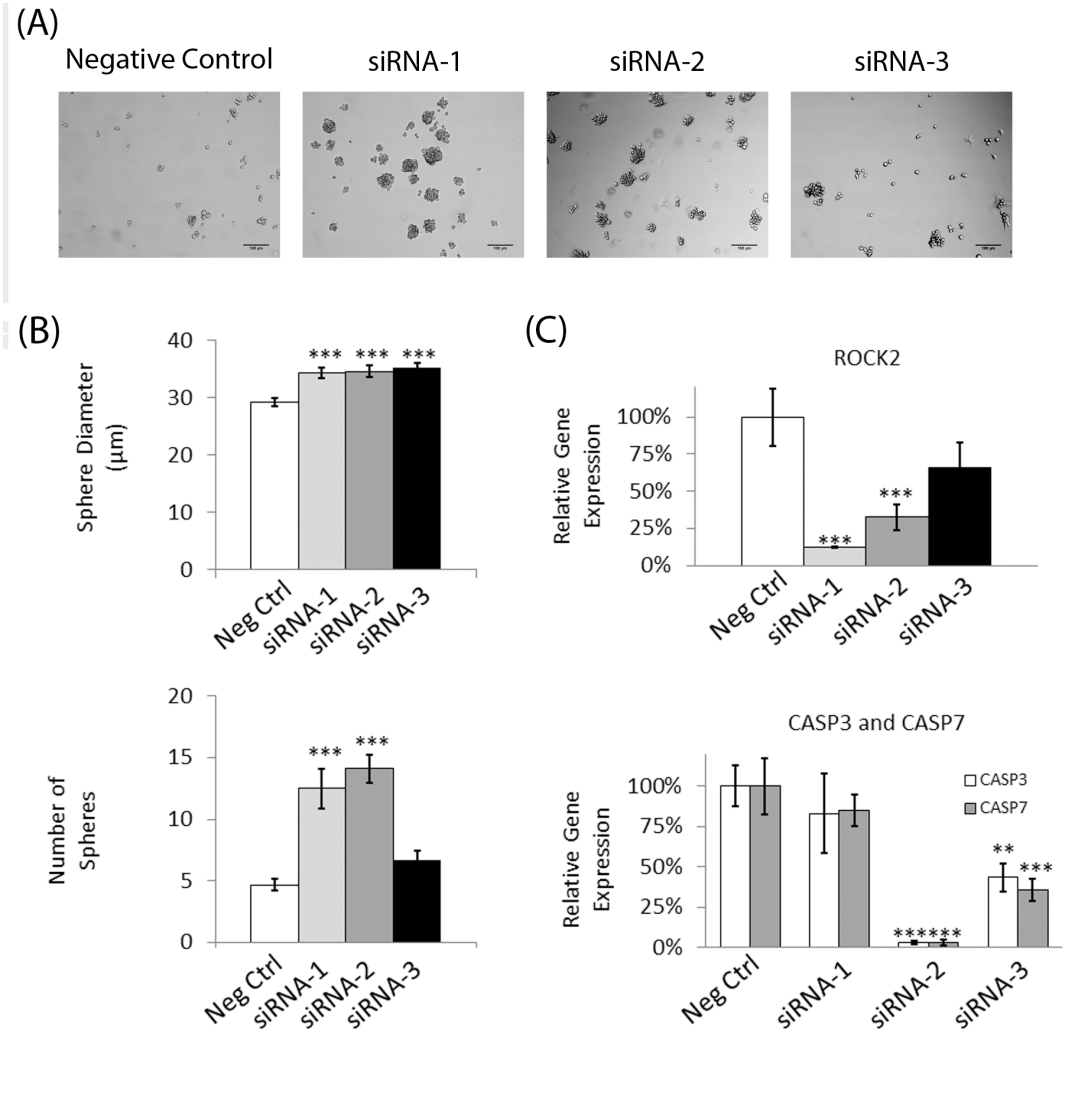
Knockdown of ROCK2 shows similar behavior to Y-27632 and Fasudil. U87-MG cells were transfected with ROCK2 siRNA and grown as tumorspheres for 3 days. The cells’ ability to form spheres was analyzed, and qRT-PCR was performed to confirm the success of the transfection. A) Representative micrographs of each experimental group on Day 1 (Scale bar = 100 μm). B) The sphere diameter (mean ± SE; *n* = 100; * *p* < 0.05, ** *p* < 0.01, and *** *p* < 0.001) and number of spheres per field of view were analyzed for all experimental groups on Day 1 (mean ± SE; *n* = 20; * *p* < 0.05, ** *p* < 0.01, and *** *p* < 0.001). C) qRT-PCR was performed on the transfected cells to confirm their gene expression levels of *ROCK2*, *CASP3*, and *CASP7*. Expression is reported as percentage of that of negative control (mean ± SE; *n* = 3; * *p* < 0.05, ** *p* < 0.01, and *** *p* < 0.001).

In order to exclude the possibility of off-target effects of the ROCK inhibitors, RNA interference via small interfering RNA (siRNA) was employed. *ROCK2* was targeted as it is a commonly found ROCK pathway gene in brain cells (10). Sphere formation and size analysis on the knockdown cells was performed as before. Knockdown of *ROCK2* increased the quantity of tumorspheres formed compared to negative control (*p* < 0.05) as it did with Y-27632 and fasudil (Fig 6). Successful gene silencing of *ROCK2* was validated via qRT-PCR. Interestingly, we also observed a down-regulation of apoptosis genes *CASP3* and *CASP7*. Thus we confirmed that silencing of *ROCK2* led to decreased apoptosis as we previously observed with Y-27632 and fasudil ROCK inhibitors. These data further confirm that the phenotypic changes we observed from Y-27632 and fasudil treatments were not due to off-target effects of the drugs.

## Discussion

To date, the effect of ROCK inhibitors on glioblastoma cells has been largely under characterized. There have been conflicting reports as to whether inhibition of the ROCK pathway inhibits or induces apoptosis in these cells [25, 28, 29]. Our data showed that ROCK inhibitors Y-27632 and fasudil in sublethal concentrations (<100 μM) inhibited apoptosis in established GBM cell line (U87-MG), in patient-derived GBM xenoline (JX12), and in primary GBM cell line (SMC448; Figs 1 and 2). Since cells undergo a great deal of stress when grown *in vitro*, protection from apoptosis provided by ROCK inhibitors allows for a greater cell yield. In addition, Y-27632 and fasudil encouraged tumorsphere formation. Tumorspheres were enriched for GSC-like cells; thus, the increased number of tumorspheres signified increased numbers of GSC-like cells. Our data showed that Y-27632 and fasudil increase the number of cells expressing the GSC marker SOX2 in U87-MG and JX12 and allowed for faster sphere generation in LDAs in all three cell lines. Others have shown that ROCK inhibition leads to cytoskeletal and pro-survival responses in non-cancerous astrocytes [26]. This corroborates with our results, and indicates that supplementation of GSC culture media with ROCK inhibitors may be a promising new technique for propagation of GSC-like cells *in vitro*.

Cells grown *in vitro* are subjected to many types of unnatural stresses, the most common of which is shear stress during passaging. When cells are grown in three-dimensional tumorsphere culture, they must be dissociated into single cells periodically to encourage further expansion and prevent necrosis within the spheres. The most common method for dissociation is trituration, which mechanically separates the cells through shear forces. However, trituration can be very stressful and even fatal to the cells. Our data suggested that ROCK inhibitors helped maintain more GSC-like cells by protecting them from this dissociation-induced shear stress (Figs 2 and 3). Treatment of cells with Y-27632 or fasudil decreased the amount of cell death during this stressful process and increased the cell yield.

Previous studies showed that GBM cells grown as tumorspheres in serum-free GSC-enriching media retained a high level of tumorigenicity *in vivo* [3, 11]. Additional studies indicated that the GSC marker SOX2 is necessary to initiate tumor growth [30–32]. These studies thus suggest that *in vitro* tumorsphere formation and presence of SOX2-positive cells are apt predictors of *in vivo* tumorigenicity. While future studies will need to directly confirm the role of ROCK inhibition on increasing *in vivo* tumorigenic potential of GBM cells, our results suggest that ROCK inhibition may be one of the mechanisms that results in an enrichment of GSC/BTIC-like cells *in vivo*.

GSCs/BTICs are very important for understanding the aggressive nature and characteristics of glioblastoma. Currently, very little is known about these cells because they account for such a small subpopulation of glioblastoma cancer cells. In order to grow larger numbers of these cells for research purposes, some labs have started large-scale *ex vivo* expansion in small animals [33, 34]. This, however, is a labor-intensive, expensive, poorly controlled, and slow-throughput method of GSC expansion. Some efforts are thus being made by us and others to use bioreactor systems for the propagation of the cancer stem cells. These bioreactors allow for faster propagation of GSC/BTIC-like cells but have the potential disadvantage of exposing the cells to nearly constant shear stress. Shear stress, depending on the amount, can be fatal to cells and can lead to low viability of cells when grown in bioreactors. We proposed that ROCK inhibitors would help cells survive the stressful environment in bioreactors by inhibiting apoptosis. The inhibitors would decrease the susceptibility of cells to shear stress from agitators and could lead to increased cell viability and cell yield. Our results suggested that the addition of ROCK inhibitors to GSC media could lead to enhanced expansion of GSC-like cells in bioreactors. Furthermore, Y-27632 was previously reported to trigger dedifferentiation to increase stem cell population [35]. Our data here as well as preliminary data with other cells (MDA-MB-231 and MCF-7; data not shown) are in support of this hypothesis and indicated that ROCK inhibitors could be useful for culturing not only GSC-like cells but other types of cancer stem cells as well.

In addition, ROCK inhibitors could be useful in understanding the cells’ response to physiological shear stresses. While glioblastoma rarely metastasizes, the results from this study could have important implications for metastasis. During metastasis, cells enter the blood stream and are subjected to very high levels of shear stress. Cancer cells, especially cancer stem cells, have been found to have increased resistance to the shear stresses of the blood stream. If inhibition of the ROCK pathway inhibits apoptosis, manipulation of this pathway could be used to target metastasizing cancer cells such as breast cancer cells [20].

Addition of ROCK inhibitors to GSC media allows for more efficient culturing of GSC-like cells. These compounds seem to have very few adverse effects on the cells and allow for increased cell viability, sphere formation, and GSC propagation. Supplementation of GSC culture media could prove to be a very promising technique for improved *in vitro* culture.
